# Complete Genome Sequence of *Pseudomonas* sp. UK4, a Model Organism for Studies of Functional Amyloids in *Pseudomonas*

**DOI:** 10.1128/genomeA.00898-14

**Published:** 2014-09-11

**Authors:** Morten Simonsen Dueholm, Heidi Nolsøe Danielsen, Per Halkjær Nielsen

**Affiliations:** Center for Microbial Communities, Department of Biotechnology, Chemistry, and Environmental Engineering, Aalborg University, Aalborg, Denmark

## Abstract

Here, we present the complete genome of *Pseudomonas* sp. UK4. This bacterium was the first *Pseudomonas* strain shown to produce functional amyloids, and it represents a model organism for studies of functional amyloids in *Pseudomonas* (Fap).

## GENOME ANNOUNCEMENT

Members of the gammaproteobacterial genus *Pseudomonas* are Gram-negative, rod shaped, bacteria renowned for their incredible metabolic capacity, physiologic versatility, and ability to form biofilms, which allows them to occupy a wide range of environmental niches (1). *Pseudomonas* sp. UK4 was originally isolated from a biofilm formed in a drinking water reservoir in a random search for bacteria producing functional amyloids (2, 3). UK4 was taxonomically assigned to the *P. fluorescens* group based on 16S rRNA gene nucleotide sequence analysis, as well as physiological and biochemical features (4). UK4 was shown to produce functional amyloid fimbriae, which were distinct from the previously known *curli* fimbriae of *E. coli* (5–7). *Pseudomonas* sp. UK4 represents an important model organism for studies of functional amyloids in *Pseudomonas* (Fap)*.*

Genomic DNA was isolated using the PowerMicrobial Maxi DNA isolation kit (MoBIO, Carlsbad, CA). Paired-end and mate-pair libraries were prepared with the TruSeq DNA PCR-Free and mate-pair (v2) sample preparation kits (Illumina, Germany), respectively. The mate-pair library was prepared without any size selection. All procedures were carried out as recommended by the manufacturer. Sequencing of the libraries was performed using a MiSeq sequencer (Illumina, Germany). The paired-end reads were trimmed for adapters and quality using the build-in tool of CLC Genomics Workbench v7.0 (CLC bio, USA). The mate-pair reads were trimmed for adapters and quality using the NextClip tool v0.8 (8). The genome was *de novo* assembled from the paired-end and mate-pair data using SPAdes genome assembler v3.1.0 (9) with k-mers of 55, 77, 99, and 127 bp. Manual scaffolding of contigs was carried out based on paired-end and mate-pair information. Cytoscape v2.8.3 (10) was used for visualization and manual inspection of the assemblies as described elsewhere (11). Gaps were closed and subsequent validated by manual read mapping in CLC Genomics Workbench. The average coverage of the assembly was 150×. Annotation was done using the NCBI prokaryotic genome automatic annotation pipeline (PGAAP) (12).

The complete genome of *Pseudomonas* sp. UK4 is composed of a circular chromosome of 6,064,456 bp. The overall G+C content is 60.1%. The strain is most closely related to *Pseudomonas* sp*.* strain TKP, with which it shares 84.8% average nucleotide identity (ANIb) (13, 14). Annotation by the NCBI PGAAP identified 5,178 coding sequences (CDS) as well as 19 rRNA (5S, 16S, or 23S) and 68 tRNA genes.

### Nucleotide sequence accession number.

This whole-genome sequencing project has been deposited at GenBank under the accession no. CP008896.

## References

[1] Silby MW Winstanley C Godfrey SAC Levy SB Jackson RW 2011 *Pseudomonas* genomes: diverse and adaptable. FEMS Microbiol. Rev. 35:652–680. 10.1111/j.1574-6976.2011.00269.x 21361996

[2] Larsen P Nielsen JL Dueholm MS Wetzel R Otzen D Nielsen PH 2007 Amyloid adhesins are abundant in natural biofilms. Environ. Microbiol. 9:3077–3090. 10.1111/j.1462-2920.2007.01418.x 17991035

[3] Dueholm MS Petersen SV Sønderkaer M Larsen P Christiansen G Hein KL Enghild JJ Nielsen JL Nielsen KL Nielsen PH Otzen DE 2010 Functional amyloid in *Pseudomonas*. Mol. Microbiol. 77:1009–1020. 10.1111/j.1365-2958.2010.07269.x 20572935

[4] Garrity G Brenner DJ Krieg NR Staley JR 2007 Bergey’s manual of systematic bacteriology, vol 2 The proteobacteria, part B: the gammaproteobacteria. Springer Verlag Science & Business Media, Berlin

[5] Chapman MR Robinson LS Pinkner JS Roth R Heuser J Hammar M Normark S Hultgren SJ 2002 Role of *Escherichia coli* curli operons in directing amyloid fiber formation. Science 295:851–855. 10.1126/science.1067484 11823641 PMC2838482

[6] Dueholm MS Albertsen M Otzen D Nielsen PH 2012 Curli functional amyloid systems are phylogenetically widespread and display large diversity in operon and protein structure. PLoS One 7:e51274. 10.1371/journal.pone.0051274 23251478 PMC3521004

[7] Dueholm MS Otzen D Nielsen PH 2013 Evolutionary insight into the functional amyloids of the pseudomonads. PLoS One 8:e76630. 10.1371/journal.pone.0076630 24116129 PMC3792158

[8] Leggett RM Clavijo BJ Clissold L Clark MD Caccamo M 2014 NextClip: an analysis and read preparation tool for Nextera long mate pair libraries. Bioinformatics 30:566–568. 10.1093/bioinformatics/btt702 24297520 PMC3928519

[9] Bankevich A Nurk S Antipov D Gurevich AA Dvorkin M Kulikov AS Lesin VM Nikolenko SI Pham S Prjibelski AD Pyshkin AV Sirotkin AV Vyahhi N Tesler G Alekseyev MA Pevzner PA 2012 SPAdes: A new genome assembly algorithm and its applications to single-cell sequencing. J. Comput. Biol. 19:455–477. 10.1089/cmb.2012.0021 22506599 PMC3342519

[10] Smoot ME Ono K Ruscheinski J Wang P-L Ideker T 2011 Cytoscape 2.8: new features for data integration and network visualization. Bioinformatics 27:431–432. 10.1093/bioinformatics/btq675 21149340 PMC3031041

[11] Albertsen M Hugenholtz P Skarshewski A Nielsen KL Tyson GW Nielsen PH 2013 Genome sequences of rare, uncultured bacteria obtained by differential coverage binning of multiple metagenomes. Nat. Biotechnol. 31:533–538. 10.1038/nbt.2579 23707974

[12] Angiuoli SV Gussman A Klimke W Cochrane G Field D Garrity GM Kodira CD Kyrpides N Madupu R Markowitz V Tatusova T Thomson N White O 2008 Toward an online repository of standard operating procedures (SOPs) for (meta)genomic annotation. Omics 12:137–141. 10.1089/omi.2008.0017 18416670 PMC3196215

[13] Richter M Rosselló-Móra R 2009 Shifting the genomic gold standard for the prokaryotic species definition. Proc. Natl. Acad. Sci. U. S. A. 106:19126–19131. 10.1073/pnas.0906412106 19855009 PMC2776425

[14] Ohtsubo Y Kishida K Sato T Tabata M Kawasumi T Ogura Y Hayashi T Tsuda M Nagata Y 2014 Complete genome sequence of *Pseudomonas* sp. strain TKP, isolated from a γ-hexachlorocyclohexane-degrading mixed culture. Genome Announc. 2(1):e01241-13. 10.1128/genomeA.01241-13 24482516 PMC3907731

